# Supplementary material for: Phosphoregulation of the novel hemi-arrestin MAPK scaffold Sms1 prevents untimely mating
Source: Nat Commun. 2026 Mar 17;17:4084. doi: 10.1038/s41467-026-70631-9 (PMC13144348; doi:10.1038/s41467-026-70631-9)
Supplement: Supplementary file 1 — Supplementary information [file 41467_2026_70631_MOESM1_ESM.pdf]

Supplementary information

for

Phosphoregulation of the novel hemi-arrestin MAPK scaffold Sms1 prevents untimely mating

Boris Sieber[1]*, Laura Merlini[1], Wanlan Li[1], Maëlys Besomi[1], Laetitia Michon[1], Sushila Gordon-Lennox[1] and Sophie G Martin[1,2]*

[1]Department of Molecular and Cellular Biology, University of Geneva

*Authors for correspondence: Sophie.Martin@unige.ch (SGM); Boris.Sieber@unige.ch (BS)
[2]Lead contact

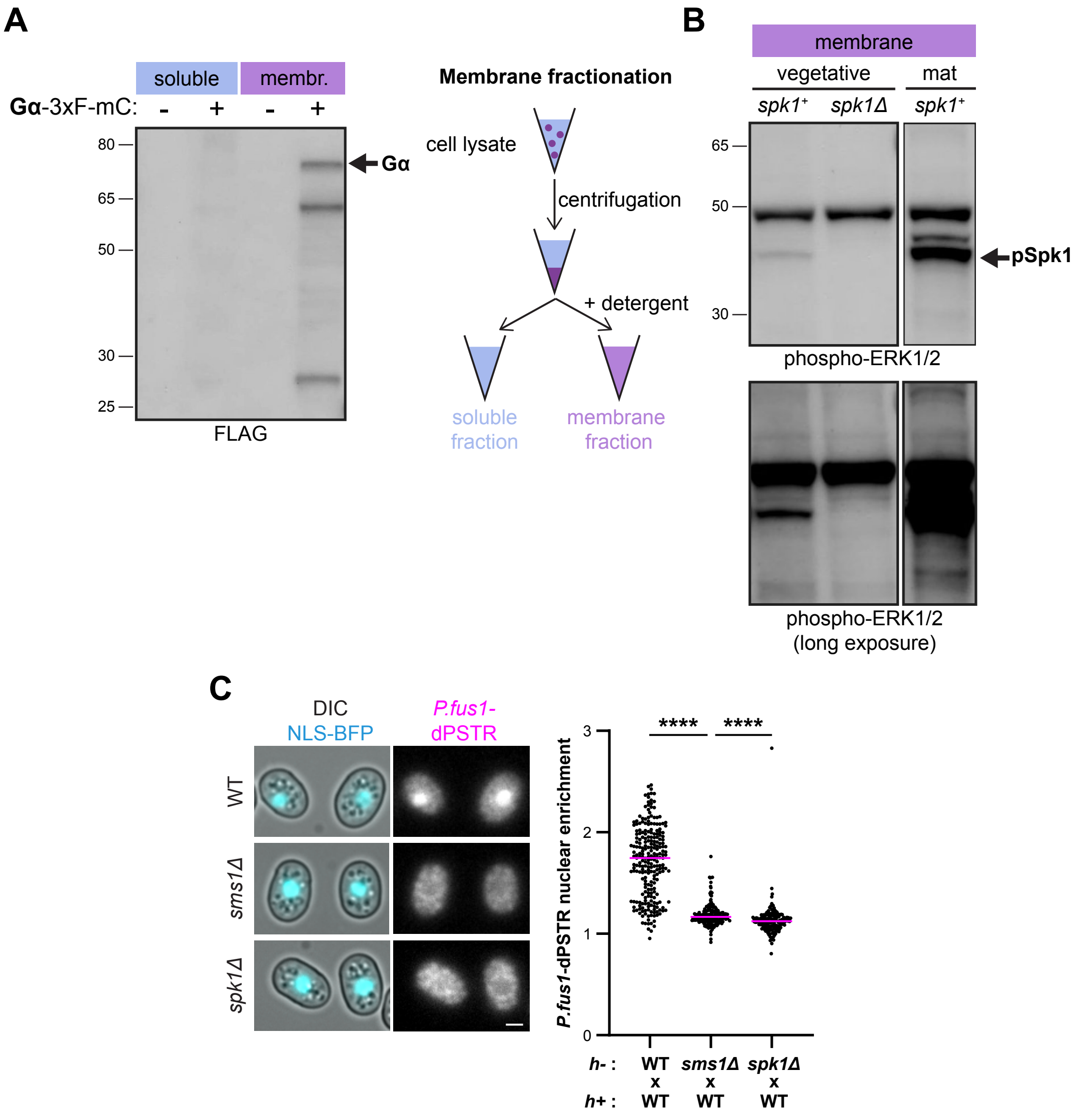

Supplementary Fig. 1: Controls for membrane fractionation, phospho-Spk1 antibody specificity and dPSTR assay

A. Membrane and soluble fractions from cells over-expressing Gpa1-3xFLAG-mCherry under the *nmt41* promoter immunoblotted for FLAG. The arrow marks the position of the $G\alpha^{Gpa1}$. **B.** Membrane fractions from *h90* cells deleted for spk1 (*spk1Δ*) or expressing *spk1*^{as2} (*spk1+*) in rich MSL+N medium (vegetative) and MSL-N starvation medium (induced to mate; mat) immunoblotted for phosphorylated ERK1/2. The arrow marks the specific phospho-Spk1 signal. **C.** Nuclear enrichment of the dPSTR reporter in *h-* WT, *sms1Δ* and *spk1Δ* cells crossed with *h+* WT and imaged after 24 hrs. Unmated cells were chosen for analysis. ****P<0.0001. N >190 cells for two independent experiments. Scale bar: 2µm.

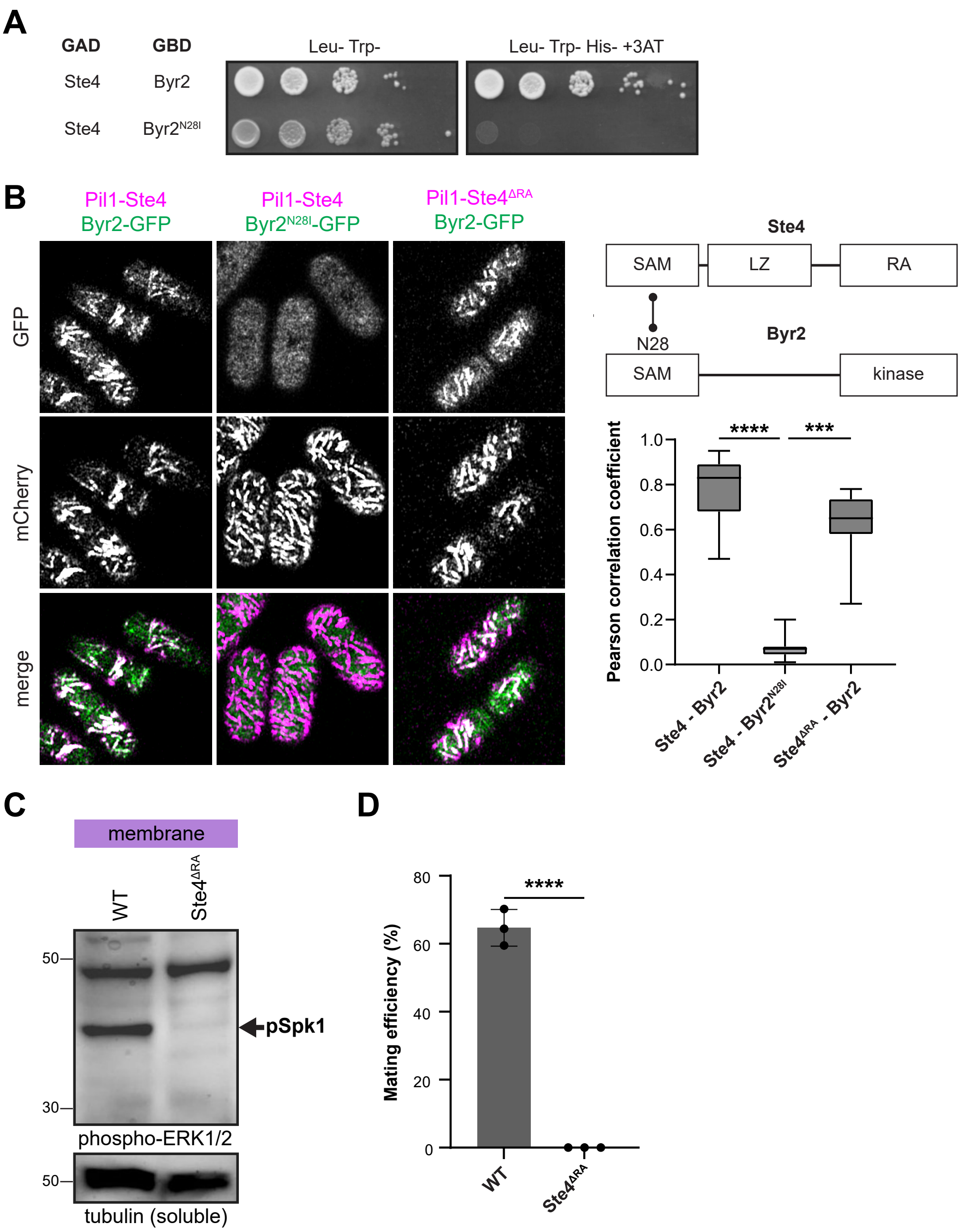

Supplementary Fig. 2: Controls for Pil1 and two-hybrid assays, and analysis of *ste4$^{\Delta RA}$* mutant

A. Yeast two-hybrid assay of Ste4 with Byr2, blocked by Byr2^{N28I} (Tu et al., 1997). **B.** Colocalisation of Pil1-Ste4-mCherry (WT or ΔRA) with Byr2-GFP (WT or N28I). Pearson correlation coefficient values are shown on the right ($n \geq 5$ cells for three independent experiments). ****P*=0.0004; *****P*<0.0001. Scale bar: 2µm. **C.** Phospho-Spk1 (arrow) in membrane fractions of Ste4-GFP and Ste4$^{\Delta RA}$-GFP cells (both with also tagged Sms1-sfGFP) in MSL-N starvation induced conditions. Tubulin serves as loading control. **D.** Mating efficiency of *h90* WT and *ste4$^{\Delta RA}$-GFP* cells ($n \geq 500$ cells for three independent experiments) with error bars as s.d. *****P*<0.0001.

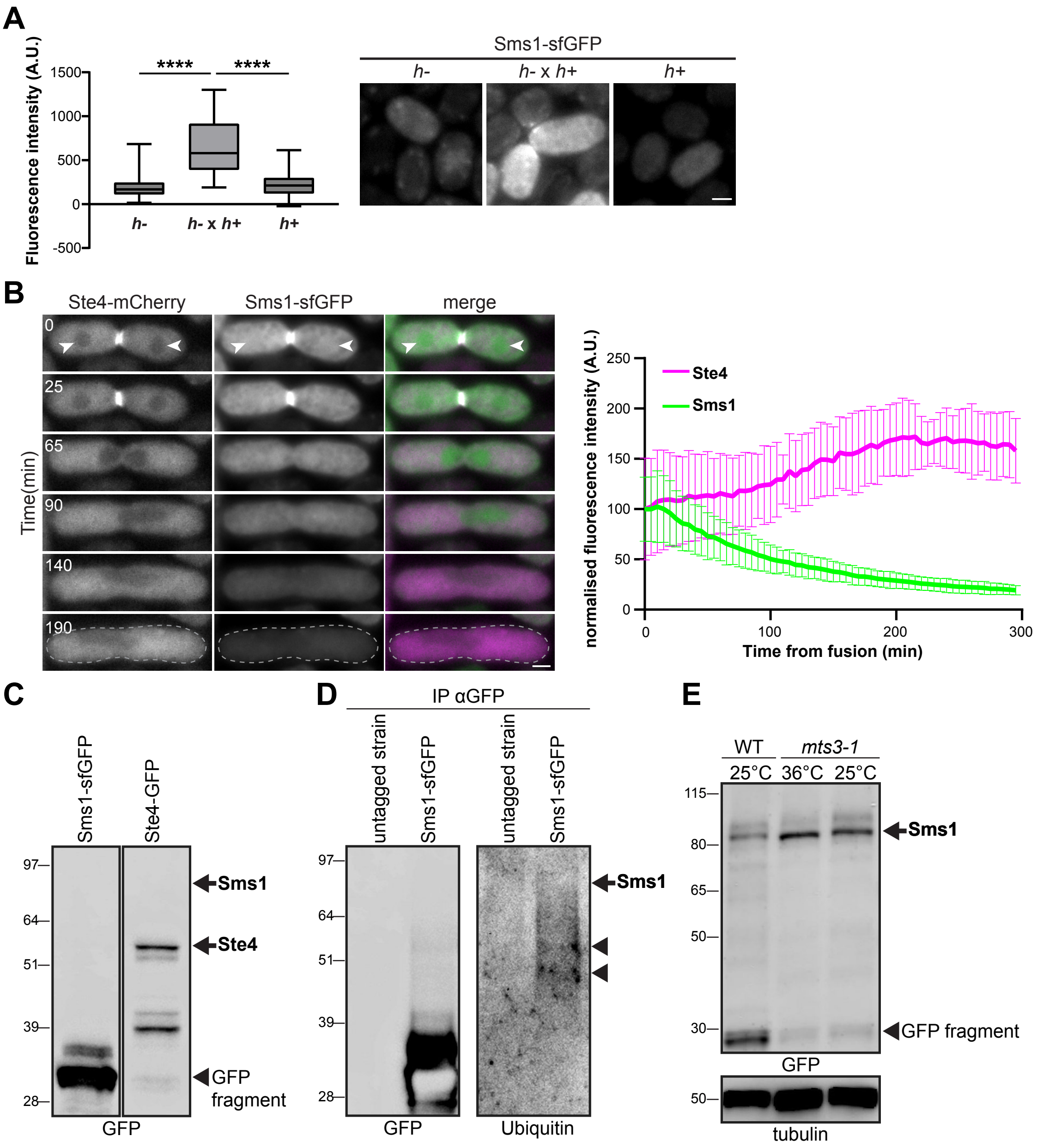

Supplementary Fig. 3: Sms1 is an unstable protein expressed specifically during mating

A. Whole-cell fluorescence intensity of Sms1-sfGFP upon nitrogen starvation (*h*- and *h*+) and mating (*h*- x *h*+) after subtraction of the autofluorescence (*n*>30 cells). *****P*<0.0001. **B.** Timelapse of Sms1-sfGFP and Ste4-mCherry in *h90* mating cells and early zygote. Note the nuclear depletion of Ste4 (arrowheads) but not of Sms1, and the rapid decrease in Sms1 (but not Ste4) levels in the zygote. Whole-zygote fluorescence intensity of Sms1-sfGFP and Ste4-mcherry, normalised to t0, is shown on the right (*n*=14 cells), with error bars as s.d. **C.** Whole cell extracts from *h90* cells expressing endogenously tagged Ste4-GFP or Sms1-sfGFP in MSL-N starvation conditions immunoblotted for GFP. **D.** GFP-based immunoprecitipates from *h90* endogenously tagged Sms1-sfGFP or untagged cells immunoblotted for ubiquitin. Arrowheads indicate ubiquitinated fragments of Sms1-sfGFP. **E.** Increased stability of Sms1-sfGFP expressed under *nmt41* promoter in hypomorphic *mts3-1* proteasome mutant at the indicated temperature during 3h. In (A.B), scale bar: 2µm. In (C-E), arrows show the position of full-length proteins.

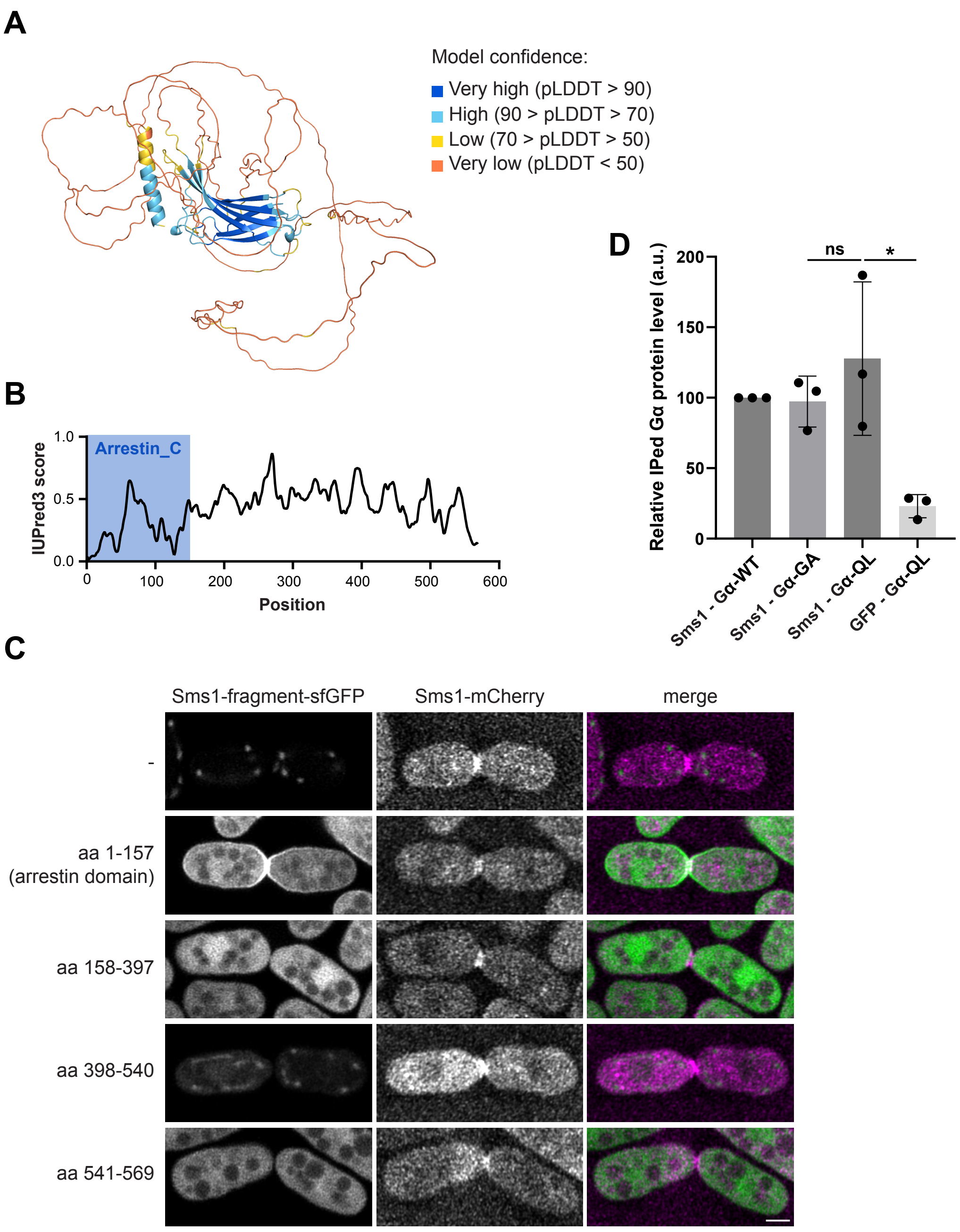

Supplementary Fig. 4: Sms1 predicted structure and fragment localisation

A. Structural model of Sms1 predicted by AlphaFold2 with predicted local distance difference test (pLDDT) score as confidence measure. **B.** Disorder scores of Sms1 generated using IUPred3. **C.** Colocalisation of sfGFP-tagged Sms1 fragments expressed under *nmt41* promoter with endogenous full-length Sms1-mCherry in mating cells. Scale bar: 2μm **D.** Gα levels from GFP immunoprecipitations were quantified relative to total Gα level (input) from three biological replicates (as in Fig 4H). In each replicate, the relative immunoprecipitated Gα level was normalised to Gα-WT. Error bars represent s.d. **P*=0.0177; ns, not significant (P=0.7592).

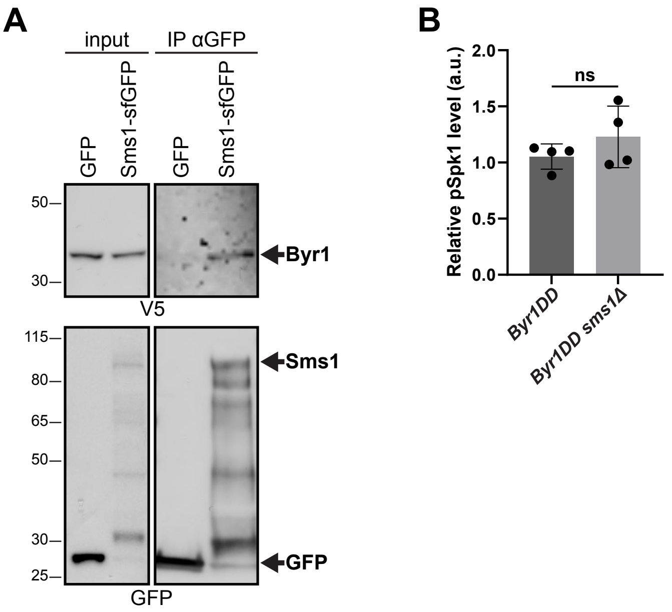

Supplementary Fig. 5: Sms1 interaction with Byr1 and effect on pSpk1 in *byr1*^{DD} cells

A. V5-Byr1 co-precipitates with Sms1-sfGFP. Whole cell extracts from vegetative cells expressing V5-Byr1 and Sms1-sfGFP or GFP under *nmt41* promoter were immunoblotted for V5 and GFP. **B.** Phospho-Spk1 levels in *h90 byr1*^{DD} and *h90 byr1*^{DD} *sms1Δ* cells were quantified relative to tubulin level from four biological replicates. Error bars represent s.d. ns, not significant (P=0.2825).

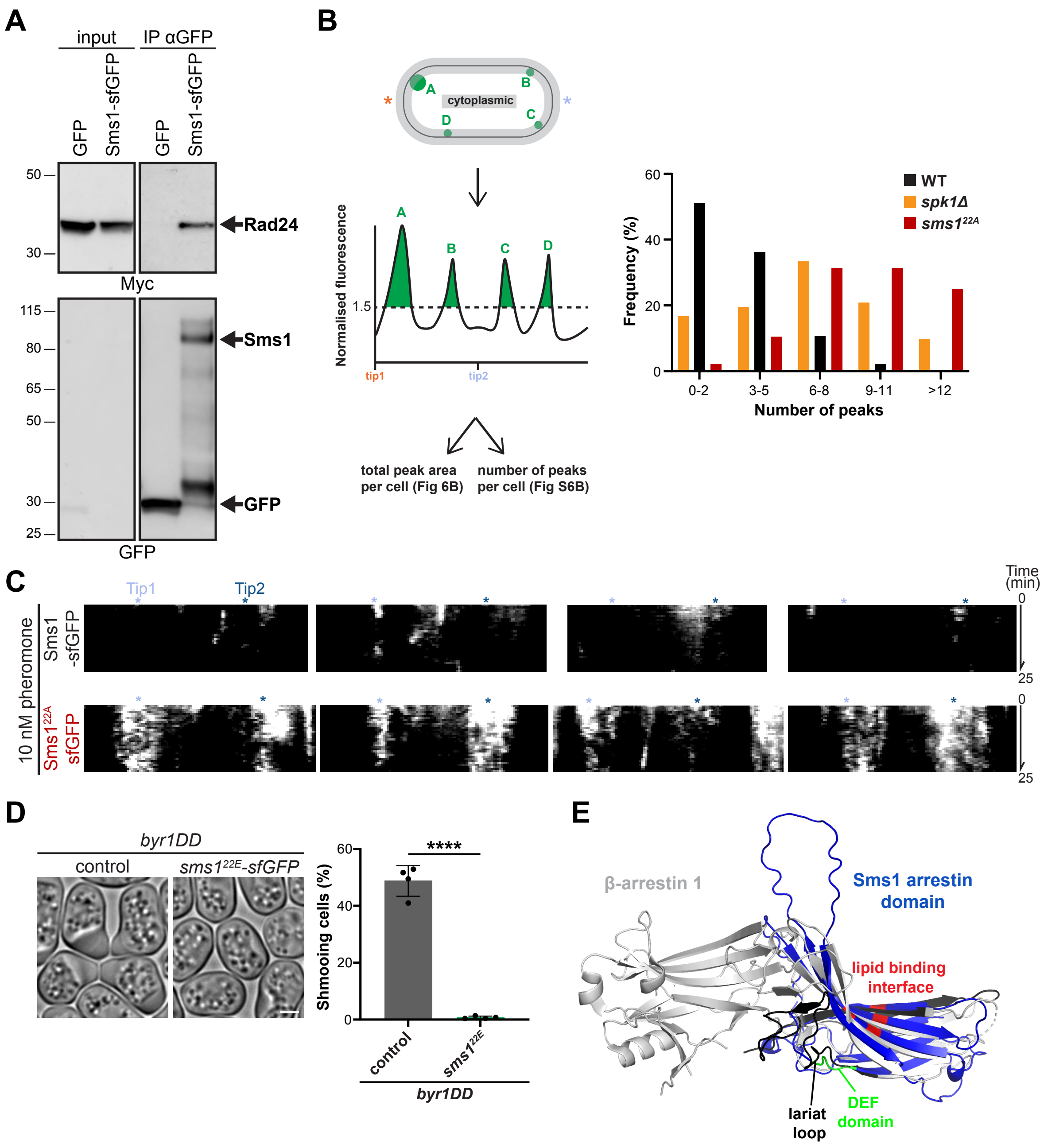

Supplementary Fig. 6: Phosphorylation of Sms1 inhibits its function

A. 14-3-3 co-precipitates with Sms1-sfGFP. Whole-cell extracts from vegetative cells expressing Rad24-3xMyc and Sms1-sfGFP or GFP as a control under *nmt41* promoter were immunoprecipitated with anti-GFP beads and immunoblotted for Myc and GFP. **B.** Quantification strategy and histogram distribution of the number of native Sms1^{22A}-sfGFP or Sms1-sfGFP membrane patches in *h*+ starved cells (*n*>40 cells) defined as the number of peaks per cell above the baseline level in cells as in Fig. 6B. **C.** Additional kymographs from timelapse imaging of Sms1-sfGFP and Sms1^{22A}-sfGFP in *h- sxa2Δ* cells stimulated with 10nM P-factor for 60 min, as in Fig. 6C. Identical contrasting parameters were used for both conditions, with asterisks indicating cell poles. **D.** DIC pictures and shmooing efficiency of *h90 byr1^{DD}* with *sms1+* or *sms1^{22E}-sfGFP* at endogenous locus as in Fig. 5G. n> 500 cells for four independent experiments. Error bars

represent s.d. ****$P<0.0001$. Scale bar: 2μm. **E.** Sequence-independent structural alignment of the Alphafold2 prediction of Sms1 arrestin domain (blue) with the X-ray crystal structure of bovine β-arrestin 1 (ID: 1G4R) with a root mean square deviation (RMSD) of 3.406 Å. MAP kinase interfaces identified in [1] are shown in black. On the convex side of Sms1 arrestin domain the canonical DEF MAPK-docking motif is highlighted in green, whereas the basic residues on the concave side are in red.

Supplementary Table 1: List of the 20 most enriched Ste4 interaction partners identified by mass spectrometry.

Uniprot ID	Protein name	Gene	q-value	fold change
**P78761**	ubiquinol-cytochrome-c reductase complex core protein Qcr2	qcr2	2.99E-03	1309.89
**P28829**	MAP kinase kinase kinase Byr2	byr2	1.74E-03	619.84
**O74313**	Schizosaccharomyces specific protein	SPBC15D4.08c	1.96E-03	231.86
**Q10482**	vacuolar amino acid transmembrane transporter Stm1	stm1	1.74E-03	120.65
**O74966**	Sm snRNP core protein Smg1	smg1	1.67E-03	88.39
**O59778**	biotin synthase	bio2	6.29E-04	82.71
**O13752**	TLC domain-containing protein Tlc4	tlc4	9.02E-03	71.66
**Q10136**	pheromone-MAPK scaffold Sms1	sms1	1.86E-03	66.62
**O74433**	ubiquinol-cytochrome-c reductase complex subunit 9	qcr9	3.84E-02	57.88
**Q09154**	ubiquinol-cytochrome-c reductase complex subunit 5, Rieske iron-sulfur protein, Rip1	rip1	6.93E-03	54.38
**O14344**	IMP dehydrogenase Gua1	gua1	3.79E-03	50.63
**P04551**	cyclin-dependent protein kinase Cdk1/Cdc2	cdc2	8.39E-04	47.90
**Q9USY1**	bifunctional pseudouridylate synthase/pseudouridine kinase	SPBC1861.05	2.20E-02	46.87
**O74856**	CCR4-Not complex CAF1 family ribonuclease subunit 7/8	caf1	9.26E-03	41.10
**Q10265**	Hsp70 family heat shock protein Ssa1	ssa1	1.12E-03	37.46
**O43028**	vacuolar protein-membrane adaptor Vac8	vac8	7.14E-03	36.81
**Q9URZ3**	plasma membrane proline transmembrane transporter Put4	put4	7.14E-03	35.12
**O36022**	Golgi mannan polymerase I complex subunit Mnn9	mnn9	1.58E-02	34.34
**Q9UTN1**	mitochondrial carrier, oxaloacetate family anion, Oac1	oac1	1.26E-02	33.68
**O13370**	translation initiation RNA helicase Sum3	sum3	2.47E-02	33.54

Supplementary Table 2: Strain list

All strains are prototroph except otherwise indicated.

Genotype	Figure	Source	Identifier
h90 Sms1-3xFLAG-mCherry:natMX	1B	This study	YSM4387
h90 ste4Δ::hphMX	1C	This study	YSM4380
h90 wild-type	1C, 1D, 1G	Lab stock	YSM1396
h90 ste4Δ::kanMX	1D	This study	YSM4383
h90 byr2Δ::kanMX	1D	This study	YSM4409
h+ ura4+:P.act1:GFP:terminatorScAdh1:kanMX	1E	This study	YSM4329
h90 Ste4-GFP:kanMX	1E	This study	YSM4394
h+ sms1Δ::natMX6	1F	This study	YSM4366
h- sms1Δ::natMX6	1F	This study	YSM4370
h+ wild-type	1F	Lab stock	YSM1371
h- wild-type	1F, 1H	This study	YSM4393
h90 sms1Δ::natMX6	1G	This study	YSM4360
h90 ura4+:byr1Δ	1G	This study	YSM4391
h+ ade6+: P.pom1:mCherry-linker-SynZip1 ura4+: P.fus1:2xNLS-linker-SynZyp2 lys3+:P.tdh1:NLS-linker-mTagBFP2:kanMX	1H	This study	YSM4402
h+ ade6+:P.pom1:mCherry-linker-SynZip1 ura4+:P.fus1:2xNLS-linker-SynZyp2 spk1Δ::hphMX lys3+:P.tdh1:NLS-linker-mTagBFP2:kanMX	1H	This study	YSM4403
h+ ade6+:P.pom1:mCherry-linker-SynZip1 ura4+:P.fus1:2xNLS-linker-SynZyp2 lys3+:P.tdh1:NLS-linker-mTagBFP2:kanMX sms1Δ::natMX	1H	This study	YSM4408
h90 leu1+:P.nmt41:Sms1-sfGFP ade6+:P.nmt41:Ste4-3xFLAG-mCherry-hphMX ura4-D18	2A	This study	YSM4337
h90 ura4+:P.nmt41:GFP:natMX ade6+:P.nmt41:Ste4-3xFLAG-mCherry-hphMX leu1-32	2A	This study	YSM4342
h90 leu1+:P.nmt41:Pil1-Ste4-3xFLAG-mCherry ura4+:P.nmt41:Sms1ΔSBD(Δ422-433aa)-sfGFP-natMX	2C	This study	YSM4333
h90 leu1+:P.nmt41:Pil1-Ste4-3xFLAG-mCherry ura4+:P.nmt41:Sms1-sfGFP-natMX	2C	This study	YSM4334
h90 ade6+:P.nmt41:Pil1-Ste4ΔRA(Δ178-264aa)-3xFLAG-mCherry-hphMX ura4+:P.nmt41:Sms1-sfGFP:natMX leu1-32	2C	This study	YSM4348
h90 Sms1-sfGFP:hphMX	2E, 2F	This study	YSM4356
h90 Sms1ΔSBD(Δ422-433aa)-sfGFP:kanMX	2E, 2F	This study	YSM4386
h+ ura4+:P.map3:tdTomato	3A		YSM4328
h- Sms1-sfGFP:natMX scd2-mCherry:natMX	3A	This study	YSM4396
h90 Sms1-sfGFP:natMX scd2-mCherry:natMX	3B	This study	YSM4395
h90 Sms1-sfGFP:natMX Ste4-3xFLAG-mCherry:hphMX	3C	This study	YSM4355
h90 Ste4-GFP-kanMX Scd2-mCherry-natMX	3D	This study	YSM4390
h90 Sms1-sfGFP:hphMX	3E	This study	YSM4356
h90 Sms1-sfGFP:natMX ade6+:P.tdh1:NLS-GST-GBP:hphMX	3F, 3G, 3H	This study	YSM4351
h90 Sms1-sfGFP:natMX	3F, 3G, 3H	This study	YSM4352
h90 ade6+:P.tdh1:NLS-GST-GBP:hphMX	3G	This study	YSM4353
h- ura4+:P.act1-mCherry-LactC2 his5+:P.nmt41:Sms1_arrestin(aa1-157)-sfGFP:kanMX myo52-mtagBFP2:natMX	4B	This study	YSM4359
h- Myo52-mtagBFP2:natMX ura4+:P.act1:mCherry-LactC2 his5+:P.nmt41:Sms1_arrestin(aa1-157)-LBM(R40Q, K42Q, K125Q, Y132S, F134E, K142Q)-sfGFP:kanMX	4B	This study	YSM4382
h- its3-1 his+:P.nmt41:Sms1_arrestin(aa1-157)-sfGFP-kanMX ura4- leu1-	4C	This study	YSM4398
h90 Sms1-3xFLAG-mCherry:natMX his5+:P.nmt41:Sms1_arrestin(aa1-157)-sfGFP:kanMX	4C, 4F	This study	YSM4347
h90 Sms1-sfGFP:natMX	4D	This study	YSM4352
h90 sms1Δ::natMX6	4D	This study	YSM4360
h90 Sms1-LBM(R40E, K42Q, K125Q, Y132S, F134E, K142Q)-sfGFP:hphMX	4D	This study	YSM4385
h- mts3-1 ura4+:P.nmt41:Sms1-sfGFP:natMX leu1-32	4G	This study	YSM4349
h90 ura4+:P.nmt41:Gpa1-3xFLAG-mCherrySW:natMX his5+:P.nmt41:Sms1_arrestin(aa1-157)-sfGFP:kanMX	4G	This study	YSM4362
h90 ura4+:P.nmt41:Gpa1-3xFLAG-mCherrySW:natMX his5+:P.nmt41:Sms1_middle(aa158-397)-sfGFP:kanMX	4G	This study	YSM4363

h90 leu1+:P.nmt41:Sms1-sfGFP ura4+:P.nmt41:Gpa1-3xFLAG-mCherrySW-natMX	4G, 4H	This study	YSM4338
h- P.nmt1:GFP-Tna1:kanMX ura4+:P.nmt41:Gpa1-3xFLAG-mCherrySW:natMX	4H	This study	YSM4367
h90 leu1+:P.nmt41:Sms1-sfGFP ade6+:P.nmt41:Gpa1-QL(Q244L)-3xFLAG-mCherrySW:hphMX ura4-D18	4H	This study	YSM4368
h90 ura4+:P.nmt41:GFP:natMX ade6+:P.nmt41:Gpa1-QL(Q244L)-3xFLAG-mCherrySW:hphMX leu1-32	4H	This study	YSM4369
h90 leu1+:P.nmt41:Sms1-sfGFP ade6+:P.nmt41:Gpa1-GA(G242A)-3xFLAG-mCherrySW:natMX ura4-D18	4H	This study	YSM4372
h90 leu1+:P.nmt41:Sms1-sfGFP ade6+:P.nmt41:V5-Byr1DD(S214D,T218D)-hphMX ste11Δ::kanMX ura4-D18	5A	This study	YSM4346
h90 ura4+:P.nmt41:GFP:natMX ade6+:P.nmt41:V5-Byr1DD(S214D,T218D):hphMX ste11Δ::kanMX ade6+ leu1-32	5A	This study	YSM4361
h90 ura4+:P.nmt41:Ste4-GFP-natMX ade6+:P.nmt41:Spk1(R81S-M198L)-3xFLAG-mCherry-hphMX	5B	This study	YSM4339
h90 ura4+:P.nmt41:GFP:natMX ade6+:P.nmt41:Spk1(R81S-M198L)-3xFLAG-mCherry-hphMX leu1-32	5B	This study	YSM4340
h90 Ste4-GFP:kanMX	5C	This study	YSM4394
h90 Sms1-3xFLAG-mCherry:natMX	5C, 5D	This study	YSM4387
h90 Byr1-DD(S214D,T218D) sms1Δ::natMX ura4-D18	5E, 5G	This study	YSM4354
h90 Byr1DD(S214D,T218D) ura4-D18	5E, 5G	This study	YSM4392
h90 Byr1-DD(S214D,T218D) sms1Δ::natMX ura4+:P.fus1:2xNLS-linker-SynZyp2 ade6+:P.pom1:mCherry-SynZip1-termCyc1-hphMX lys3+:P.tdh1:NLS-linker-mTagBFP2 -kanMX	5F	This study	YSM4399
h90 Byr1-DD(S214D,T218D) ura4+:P.fus1:2xNLS-linker-SynZyp2 ade6+:P.pom1:mCherry-SynZip1-termCyc1:hphMX lys3+:P.tdh1:NLS-linker-mTagBFP2:kanMX	5F	This study	YSM4400
h90 Byr1-DD(S214D,T218D) ura4+:P.fus1:2xNLS-linker-SynZyp2 ade6+:P.pom1:mCherry-SynZip1:natMX lys3+:P.tdh1:NLS-linker-mTagBFP2-:kanMX spk1Δ::pfa6a-hphMX	5F	This study	YSM4405
h90 Byr1DD(S214D,T218D) ste4Δ::hphMX ura4-D18	5G	This study	YSM4371
h90 Byr1DD(S214D,T218D) spk1Δ::hphMX ura4-D18	5G	This study	YSM4377
h- ura4+:P.nmt41:Sms1-22A(T30A, S31A, S53A, S54A, T179A, T221A, T266A, S271A, T277A, S294A, S310A, S315A, T316A, S384A, S394A, S397A, S398A, S406A, S412A, S501A, S535A, S536A)-sfGFP:natMX ste11Δ::kanMX ade6-M216 leu1-32	6A	This study	YSM4374
h- ura4+:P.nmt41:Sms1-sfGFP:natMX ste11Δ::kanMX ade6-M216 leu1-32	6A	This study	YSM4375
h+ Sms1-22A(T30A, S31A, S53A, S54A, T179A, T221A, T266A, S271A, T277A, S294A, S310A, S315A, T316A, S384A, S394A, S397A, S398A, S406A, S412A, S501A, S535A, S536A)-sfGFP:hphMX	6B	This study	YSM4364
h+ Sms1-sfGFP:hphMX	6B	This study	YSM4365
h+ Sms1-sfGFP:hphMX spk1Δ::kanMX	6B	This study	YSM4404
h- Sms1-sfGFP:hphMX sxa2Δ::kanMX	6C, 6D	This study	YSM4376
h- Sms1-22A(T30A, S31A, S53A, S54A, T179A, T221A, T266A, S271A, T277A, S294A, S310A, S315A, T316A, S384A, S394A, S397A, S398A, S406A, S412A, S501A, S535A, S536A)-sfGFP-hphMX sxa2Δ::kanMX	6C, 6D	This study	YSM4397
h90 Sms1-22A(T30A, S31A, S53A, S54A, T179A, T221A, T266A, S271A, T277A, S294A, S310A, S315A, T316A, S384A, S394A, S397A, S398A, S406A, S412A, S501A, S535A, S536A)-sfGFP:hphMX	6E, 6F, 6G	This study	YSM4357
h90 Sms1-sfGFP:hphMX	6E, 6F, 6G, 6H	This study	YSM4356
h90 Sms1-22E(T30E, S31E, S53E, S54E, T179E, T221E, T266E, S271E, T277E, S294E, S310E, S315E, T316E, S384E, S394E, S397E, S398E, S406E, S412E, S501E, S535E, S536E)-sfGFP:hphMX	6G	This study	YSM4358
h90 Sms1-sfGFP:natMX	6H	This study	YSM4352
h90 Sms1-4E(T30E, S31E, S53E, S54E)-sfGFP:hphMX	6H	This study	YSM4373
h90 Sms1-4A(T30A, S31A, S53A, S54A)-sfGFP:hphMX	6H	This study	YSM4378
h90 leu1+:P.nmt41:Sms1-sfGFP ura4-D18	S1A	This study	YSM4330
h90 leu1+:P.nmt41:Sms1-sfGFP ura4+:P.nmt41:Gpa1-3xFLAG-mCherrySW-natMX	S1A	This study	YSM4338
h90 Spk1(Q119A):bsdMX	S1B		YSM4410

h90 spk1Δ::hphMX ura4+:mCherry-D4H	S1B	[2]	YSM3516
h- ade6+:P.pom1:mCherry-linker-SynZip1 ura4+:P.fus1:2xNLS-linker-SynZyp2 lys3+:P.tdh1:NLS-linker-mTagBFP2-kanMX	S1D	This study	YSM4401
h- ade6+:P.pom1:mCherry-linker-SynZip1 ura4+:P.fus1:2xNLS-linker-SynZyp2 lys3+:P.tdh1:NLS-linker-mTagBFP2:kanMX spk1Δ::hphMX	S1D	This study	YSM4406
h- ade6+:P.pom1:mCherry-linker-SynZip1 ura4+:P.fus1:2xNLS-linker-SynZyp2 lys3+:P.tdh1:NLS-linker-mTagBFP2:kanMX sms1Δ::natMX	S1D	This study	YSM4407
h+ wild-type	S1D	Lab stock	YSM1371
h90 leu+:P.nmt41:Pil1-Ste4-3xFLAG-mCherry ura4+:P.nmt41:Byr2-GFP:natMX	S2B	This study	YSM4331
h90 leu1+:P.nmt41:Pil1-Ste4-3xFLAG-mCherry ura4+:P.nmt41:Byr2-N28I-GFP-natMX	S2B	This study	YSM4332
h90 ade6+:P.nmt41-Pil1-Ste4ΔRA(Δ178-264aa)-3xFLAG-mCherry-hphMX ura4+:P.nmt41:Byr2-GFP-natMX leu1-32	S2B	This study	YSM4335
h90 Sms1-sfGFP:natMX ade6+ leu1+ ura4+ his5+	S2C	This study	YSM4352
h90 Sms1-sfGFP:natMX Ste4ΔRA(Δ178-264aa)-GFP:hphMX	S2C	This study	YSM4381
h90 Ste4ΔRA(Δ178-264aa)-GFP:hphMX	S2D	This study	YSM4384
h90 Ste4-GFP:kanMX ade6+	S2D	This study	YSM4394
h+ ura4+:P.map3:tdTomatoSms1-sfGFP:natMX	S3A	This study	YSM4350
h- wild-type	S3A	This study	YSM4393
h- Sms1-sfGFP:natMX scd2-mCherry:natMX	S3A	This study	YSM4396
h+ wild-type	S3A	Lab stock	YSM1371
h90 Sms1-sfGFP:natMX Ste4-3xFLAG-mCherry:hphMX	S3B, S3C, S3D	This study	YSM4355
h90 Sms1-3xFLAG:natMX Ste4-GFP:kanMX	S3C	This study	YSM4389
h90 wild-type	S3D	Lab stock	YSM3306
h90 leu1+:P.nmt41:Sms1-sfGFP ura4-D18	S3E	This study	YSM4330
h- mts3-1 ura4+:P.nmt41:Sms1-sfGFP:natMX leu1-32	S3E	This study	YSM4349
h90 Sms1-sfGFP:natMX ade6+:P.tdh1:NLS-GST-GBP:hphMX	S3F	This study	YSM4351
h90 Sms1-sfGFP:natMX	S3F	This study	YSM4352
h90 Sms1-3xFLAG-mCherry:natMX his5+:P.nmt41:Sms1 (aa158-397)-sfGFP-kanMX	S4C	This study	YSM4343
h90 Sms1-3xFLAG-mCherry:natMX his5+:P.nmt41:Sms1 (aa398-540)-sfGFP-kanMX	S4C	This study	YSM4344
h90 Sms1-3xFLAG-mCherry:natMX his5+:P.nmt41:Sms1 (aa541-569)-sfGFP-kanMX	S4C	This study	YSM4345
h90 Sms1-3xFLAG-mCherry:natMX his5+:P.nmt41:Sms1 (aa1-157)-sfGFP:kanMX ade6+	S4C	This study	YSM4347
h90 Sms1-3xFLAG-mCherry:natMX	S4C	This study	YSM4388
h90 leu1+:pnmt41:Sms1-sfGFP ade6+:pnmt41-V5-Byr1:hphMX ura4-D18	S5A	This study	YSM4430
h90 ura4+:pnmt41-GFP:natMX ade6+:pnmt41-V5-Byr1-hphMX leu1-32	S5A	This study	YSM4431
h90 leu1+:P.nmt41:Sms1-sfGFP ade6+:P.nmt41:Rad24-3xMyc-hphMX ura4-D18	S6A	This study	YSM4336
h90 ura4+:P.nmt41:GFP:natMX ade6+:-P.nmt41:Rad24-3xMyc-hphMX leu1-32	S6A	This study	YSM4341
h+ Sms1-22A(T30A, S31A, S53A, S54A, T179A, T221A, T266A, S271A, T277A, S294A, S310A, S315A, T316A, S384A, S394A, S397A, S398A, S406A, S412A, S501A, S535A, S536A)-sfGFP:hphMX	S6B	This study	YSM4364
h+ Sms1-sfGFP:hphMX	S6B	This study	YSM4365
h+ Sms1-sfGFP:hphMX spk1Δ::kanMX	S6B	This study	YSM4404
h- Sms1-sfGFP:hphMX sxa2Δ::kanMX	S6C	This study	YSM4376
h- Sms1-22A(T30A, S31A, S53A, S54A, T179A, T221A, T266A, S271A, T277A, S294A, S310A, S315A, T316A, S384A, S394A, S397A, S398A, S406A, S412A, S501A, S535A, S536A)-sfGFP-hphMX sxa2Δ::kanMX	S6C	This study	YSM4397
h90 Byr1DD(S214D, T218D) Sms1-22E(T30E, S31E, S53E, S54E, T179E, T221E, T266E, S271E, T277E, S294E, S310E, S315E, T316E, S384E, S394E, S397E, S398E, S406E, S412E, S501E, S535E, S536E)-sfGFP:hphMX ura4-D18	S6D	This study	YSM4379
h90 Byr1DD(S214D, T218D) ura4-D18	S6D	This study	YSM4392

Supplementary Table 3: List of antibodies and magnetic beads

Reagent	Source	Identifier
Rabbit monoclonal anti-phospho-p44/42 MAPK (Erk1/2) (Thr202/Tyr204) (D13.14.4E)	Cell Signaling Technology	Cat#4370; RRID:AB_2315112
Mouse monoclonal anti-alpha tubulin HRP conjugated	Abcam	Cat#ab40742; RRID:AB_880625
Mouse monoclonal anti-beta actin	Abcam	Cat#ab8224; RRID: AB_449644
Mouse monoclonal anti-FLAG (M2)	Sigma	Cat#F1804; RRID:AB_262044
Mouse monoclonal anti-GFP	Roche	Cat#11814460001; RRID:AB_390913
Mouse monoclonal anti-ubiquitin (P4D1)	Cell Signaling Technology	Cat#3936; RRID:AB_331292
Mouse monoclonal anti-V5 HRP conjugated	Invitrogen	Cat#R961-25; RRID:AB_2556565
Goat polyclonal anti-Myc	Abcam	Cat#ab9132; RRID:AB_307033
Goat polyclonal anti-rabbit HRP conjugated	Promega	Cat#W401B; RRID:AB_430833
Goat polyclonal anti-mouse HRP conjugated	Promega	Cat#W402B; RRID:AB_430834
Mouse monoclonal anti-goat HRP conjugated	Santa Cruz	Cat#sc-2354; RRID:AB_628490
Magnetic agarose anti-GFP	ChromoTek	Cat#gtma-20; RRID:AB_2631358
Magnetic agarose Anti-DYKDDDDK(FLAG)	ThermoFisher	Cat#A36797; RRID:AB_3106985
Magnetic Bead Conjugate anti-phospho-p44/42 MAPK (Erk1/2) (Thr202/Tyr204) (D13.14.4E)	Cell Signaling Technology	Cat#9488; RRID:AB_10898026

Supplementary Table 4: List of software and algorithms

Software and algorithms	Source	Identifier
GraphPad Prism 9.5.1	GraphPad Software, LLC	RRID:SCR_002798
Fiji 2.14/1.54f	[3]	RRID:SCR_002285
PyMol 2.5.4	Schrödinger, Inc.	RRID:SCR_000305
Perseus 2.0.11.0	[4]	RRID:SCR_015753
Alphafold multimer	[5]	RRID:SCR_025453

Supplementary References

1. Qu C*, et al.* Scaffolding mechanism of arrestin-2 in the cRaf/MEK1/ERK signaling cascade. *Proc Natl Acad Sci U S A* **118**, (2021).

2. Marek M, Vincenzetti V, Martin SG. Sterol biosensor reveals LAM-family Ltc1-dependent sterol flow to endosomes upon Arp2/3 inhibition. *J Cell Biol* **219**, (2020).

3. Schindelin J*, et al.* Fiji: an open-source platform for biological-image analysis. *Nat Methods* **9**, 676-682 (2012).

4. Tyanova S*, et al.* The Perseus computational platform for comprehensive analysis of (prote)omics data. *Nat Methods* **13**, 731-740 (2016).

5. Mirdita M, Schutze K, Moriwaki Y, Heo L, Ovchinnikov S, Steinegger M. ColabFold: making protein folding accessible to all. *Nat Methods* **19**, 679-682 (2022).
