# Supplementary material for: Phosphoregulation of the novel hemi-arrestin MAPK scaffold Sms1 prevents untimely mating
Source: Nat Commun. 2026 Mar 17;17:4084. doi: 10.1038/s41467-026-70631-9 (PMC13144348; doi:10.1038/s41467-026-70631-9)
Supplement: Supplementary file 2 — Description of Additional Supplementary Files [file 41467_2026_70631_MOESM2_ESM.pdf]

**Title:** Supplementary Movie 1

**Description:** Sms1 patch dynamics and stabilisation in Sms122A

**Title:** Supplementary Movie 2

**Description:** Mating behaviour of sms122A zygotes
